# L‐Arginine and L‐Citrulline for Prevention and Treatment of Pre‐Eclampsia: A Systematic Review and Meta‐Analysis

**DOI:** 10.1111/1471-0528.18070

**Published:** 2025-01-12

**Authors:** Maureen Makama, Annie R. A. McDougall, Jenny Cao, Kate Mills, Phi‐Yen Nguyen, Roxanne Hastie, Anne Ammerdorffer, A. Metin Gülmezoglu, Joshua P. Vogel

**Affiliations:** ^1^ Women's, Children's and Adolescents' Health Program Burnet Institute Melbourne Australia; ^2^ Health and Social Care Unit, School of Public Health and Preventive Medicine Monash University Melbourne Australia; ^3^ Monash Institute of Pharmaceutical Sciences Monash University Parkville Australia; ^4^ Department of Obstetrics and Gynaecology University of Melbourne Heidelberg Australia; ^5^ Concept Foundation Geneva Switzerland

**Keywords:** hypertensive disorders of pregnancy, L‐arginine, L‐citrulline, pre‐eclampsia, pregnant women

## Abstract

**Background:**

Evidence suggests L‐arginine may be effective at reducing pre‐eclampsia and related outcomes. However, whether L‐arginine can prevent or only treat pre‐eclampsia, and thus the target population and timing of initiation, remains unknown.

**Objectives:**

To evaluate the effects of L‐arginine and L‐citrulline (precursor of L‐arginine) on the prevention and treatment of pre‐eclampsia.

**Search Strategy:**

MEDLINE, Embase, CINAHL, Global Index Medicus and the Cochrane Library were searched through 7 February 2024.

**Selection Criteria:**

Trials administering L‐arginine or L‐citrulline to pregnant women, with the comparison group receiving placebo or standard care, were included.

**Data Collection and Analysis:**

Meta‐analyses were conducted separately for prevention or treatment trials, using random‐effects models.

**Main Results:**

Twenty randomised controlled trials (RCTs) (2028 women) and three non‐randomised trials (189 women) were included. The risk of bias was ‘high’ in eight RCTs and showed ‘some concerns’ in 12. In prevention trials, L‐arginine was associated with a reduced risk of pre‐eclampsia (relative risk [RR] 0.52; 95% confidence interval [CI], 0.35, 0.78; low‐certainty evidence, four trials) and severe pre‐eclampsia (RR 0.23; 95% CI, 0.09, 0.55; low‐certainty evidence, three trials). In treatment trials, L‐arginine may reduce mean systolic blood pressure (MD −5.64 mmHg; 95% CI, −10.66, −0.62; very low‐certainty evidence, three trials) and fetal growth restriction (RR 0.46; 95% CI, 0.26, 0.81; low‐certainty evidence, two trials). Only one study (36 women) examined L‐citrulline and reported no effect on pre‐eclampsia or blood pressure.

**Conclusions:**

L‐arginine may be promising for pre‐eclampsia prevention and treatment, but findings should be interpreted cautiously. More trials are needed to determine the optimal dose and time to commence supplementation and support clinical decision‐making.

## 1 Introduction

Pre‐eclampsia affects approximately 4.6% of all pregnancies and causes > 35 000 maternal and 500 000 fetal/neonatal deaths every year [1, 2]. Pre‐eclampsia is the leading cause of iatrogenic preterm birth, responsible for up to 25% of cases [3]. Despite this, few drugs are recommended for pre‐eclampsia prevention—low‐dose aspirin (when initiated before 20 weeks gestation) and calcium supplementation (for women with low dietary intake) [4, 5]. For treatment, only antihypertensives for blood pressure (BP) control and magnesium sulphate to prevent or treat seizures have been recommended [4, 6]. Research into promising new drugs for pre‐eclampsia prevention and treatment is urgently needed [7].

In 2022, the Accelerating Innovations for Mothers (AIM) project identified L‐arginine as one of five high‐potential medicines under investigation for pre‐eclampsia prevention [8]. L‐arginine is a precursor for the endogenous synthesis of nitric oxide, a potent vasodilator that mediates vascular smooth muscle relaxation and inhibits platelet aggregation [9, 10, 11]. Abnormal placentation, dysregulation of angiogenesis, oxidative stress, and endothelial dysfunction are important aspects of the pathogenesis of pre‐eclampsia [12, 13, 14, 15, 16, 17]. Thus, the availability of L‐arginine opposes the vasoconstriction that occurs in pre‐eclampsia.

L‐arginine is a semi‐essential amino acid obtained through dietary intake (fish, meats, soy, nuts and seeds), protein turnover and endogenous synthesis from L‐citrulline [10, 18, 19]. In pregnancy, bioavailable L‐arginine levels may diminish due to increased metabolic demand resulting from fetal growth [20]. Also, when dietary L‐arginine and/or L‐citrulline intake is low, the de novo endogenous synthesis of L‐arginine from L‐citrulline cannot increase to compensate [20]. Maternal infections such as malaria may further deplete endogenous L‐arginine, making pregnant women in malaria‐endemic regions particularly vulnerable to arginine deficiency [20, 21]. Therefore, L‐arginine may be necessary to provide the substrate for nitric oxide synthesis during pregnancy [22]. Given that oral L‐citrulline, a precursor and metabolite of L‐arginine, bypasses gastrointestinal and liver metabolism and thus is more readily available in systemic circulation, L‐citrulline may improve nitric oxide bioavailability more efficiently [20, 23]. L‐arginine or L‐citrulline could be affordable and scalable interventions to improve birth outcomes, particularly in resource‐limited settings [20].

Recent reviews on L‐arginine in pregnancy have not explored the effects of L‐arginine for prevention differently from the treatment of pre‐eclampsia [24, 25, 26, 27]. This has significant clinical implications for the optimal timing of supplementation initiation and understanding which women would benefit from L‐arginine. Furthermore, no review has considered the effects of L‐citrulline during pregnancy on pre‐eclampsia. This review examines the effects of L‐arginine and L‐citrulline on prevention separately from the treatment of pre‐eclampsia and related maternal, fetal and neonatal outcomes.

## 2 Methods

The review protocol was prospectively registered in the Prospective Register of Systematic Reviews (PROSPERO) (CRD42023397474), and findings were reported following the Preferred Reporting Items for Systematic Reviews and Meta‐Analyses (PRISMA) guidelines [28].

### 2.1 Eligibility Criteria, Information Sources and Search Strategy

Randomised or non‐randomised trials that administered L‐arginine or L‐citrulline to pregnant women were eligible, regardless of formulation, route of administration, dose or regimen. Non‐randomised studies were included to provide a complete overview of the evidence base on this topic [29]. Trials that included women before the onset of pre‐eclampsia (regardless of risk) were considered prevention trials. Trials that included women diagnosed with pre‐eclampsia (according to the International Society for the Study of Hypertension in Pregnancy [ISSHP] definition) [30] were considered treatment trials. Eligible studies were those with a comparison group receiving a placebo, standard care or no treatment. Studies were excluded if the comparison group was chosen from a different population than the treatment group (e.g., if the comparison group were healthy individuals and the treatment group were not). Studies were also excluded if the comparison group received another therapeutic drug that was not concomitantly administered to the treatment group. In studies with more than one comparison group, the group that received the same interventions as the treatment group except L‐arginine/L‐citrulline was considered the comparison group. All other study designs, as well as protocols of ongoing trials, were excluded. The reference lists of reviews were hand‐searched to identify potentially eligible studies.

A comprehensive search strategy was developed with assistance from an information specialist (Appendix S1). We searched five databases from inception until 7 February 2024: MEDLINE, Embase, CINAHL, Global Index Medicus and the Cochrane Library. There were no limitations in terms of date or language of publication. Google Translate was used for studies in a language other than English. Relevant trials registered on clinicaltrials.org but not yet completed or published are summarised in Table S1.

### 2.2 Study Selection

Endnote and Covidence [31] software were used to manage citations. Two reviewers (M.M. and K.M. or J.C.) independently screened titles/abstracts and full texts of identified studies. Disagreements were resolved by discussion or consultation with another reviewer (A.R.A.M. or J.P.V.). RCTs were assessed for research integrity using an adapted assessment tool [32], which provides a transparent mechanism for identifying problematic trials to avoid misleading findings.

The primary outcome for prevention trials was the incidence of pre‐eclampsia according to the ISSHP definition [30], and for treatment trials, it was progression to pre‐eclampsia with severe features (BP ≥ 160/110 mmHg, proteinuria ≥ 5 g/24 h, presence of either HELLP syndrome, coagulation disorders or fetal growth restriction [FGR]). There were 24 secondary outcomes related to maternal, fetal and neonatal outcomes as listed in Appendix S2. Trials that reported on any of the outcomes not just the primary outcomes were included.

### 2.3 Data Extraction

Data were extracted using a standardised template. Two reviewers (M.M. and J.C./K.M.) extracted data independently, with conflicts resolved by discussion. Extracted data included author, year of publication, country, study design, population characteristics, sample size, gestational age at enrolment, dose and route of administration, use of co‐interventions, duration of use and review outcomes.

### 2.4 Assessment of Risk of Bias

Risk of bias was assessed using the revised Cochrane Risk of Bias 2 tool (RoB 2) for RCTs [33] and the Risk Of Bias In Non‐randomised Studies of Interventions tool (ROBINS‐I) for non‐randomised trials (non‐RCTs) [34]. Two reviewers (M.M. and J.C./K.M.) independently assessed the risk of bias, and disagreements were resolved through discussion or by consulting another reviewer (A.R.A.M.).

### 2.5 Data Synthesis

Random‐effects meta‐analyses using the DerSimonian and Laird method were performed using Stata SE 17 software to account for heterogeneity between studies [35, 36]. RCTs were analysed separately from non‐RCTs. Results were presented as risk ratios (dichotomous data) or mean differences (continuous data) with 95% confidence intervals (CI), and heterogeneity was estimated via *T* ^2^ and *I* ^2^ statistics. Findings were presented separately for prevention and treatment trials. The Grading of Recommendations, Assessment, Development, and Evaluations (GRADE) approach was used to assess the certainty of evidence from RCTs [37]. Two reviewers, M.M. and K.M., performed the GRADE assessment (Appendices S3 and S4). Publication bias was explored using a funnel plot asymmetry test for outcomes reported by at least 10 RCTs (Table S2 and Figure S1). Subgroup analyses were performed by indications for inclusion in trials, as defined by trial authors.

## 3 Results

**FIGURE 1:**
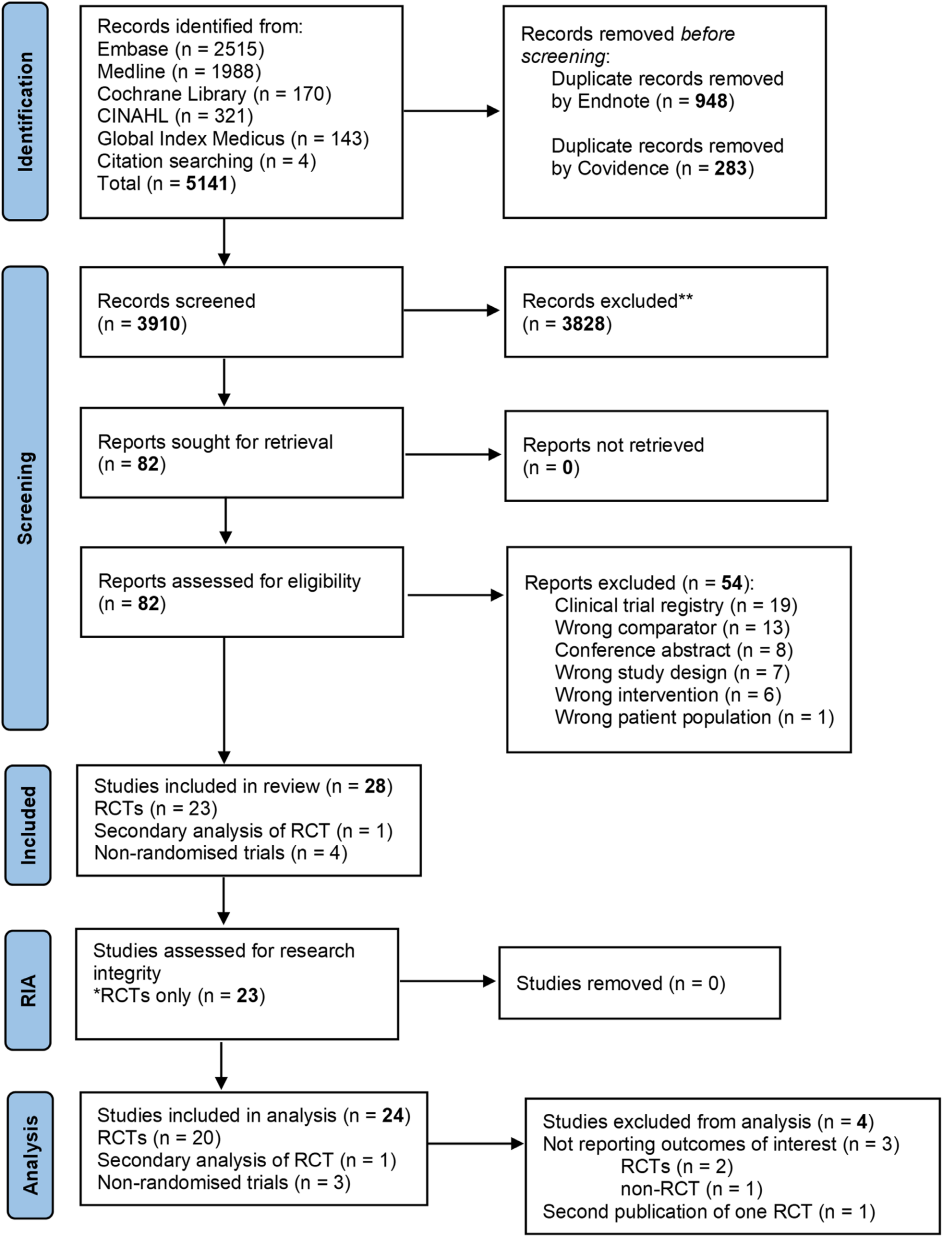
PRISMA flow chart of included studies. *Research integrity assessment tool was designed to assess randomised trials only. Non‐randomised trials did not undergo the research integrity assessment.

The database search identified 5140 studies. Four additional studies were identified by hand searching references of included studies. After removing duplicates, 3910 titles/abstracts and 82 full texts were screened. Of these, 28 studies were eligible for inclusion (Figure 1). There were 23 RCTs, one secondary analysis of an RCT [38, 39] and four non‐RCTs. Two RCTs and one non‐RCT did not report review outcomes (Table S3) [40, 41, 42]. Two studies reported on the same RCT in English and Spanish and were thus considered together [43, 44]. This resulted in 20 RCTs, one secondary analysis [38, 39] and three non‐RCTs contributing data for analysis. Only one of the RCTs reported on L‐citrulline [45]. No study was excluded following the research integrity assessment.

The 19 L‐arginine RCTs included 1992 women [38, 39, 42, 43, 44, 46, 47, 48, 49, 50, 51, 52, 53, 54, 55, 56, 57, 58, 59, 60, 61] with sample sizes ranging from 41 [60] to 672 [44] (Table S4). Trials were published from 2004 to 2024. Ten trials were in high‐income countries (France, Norway, Italy, USA, and Poland) [38, 39, 47, 48, 49, 50, 51, 53, 55, 57, 60], seven in upper‐middle‐income countries (China, Iran, Mexico and Peru) [44, 46, 52, 56, 58, 59, 62] and two in a low‐middle‐income country (India) [54, 61]. The included study populations were heterogeneous, having different indications for inclusion in the trials. The RCTs recruited women diagnosed with pre‐eclampsia (4 trials) [38, 39, 48, 55, 56]; women at high risk of pre‐eclampsia or having other hypertensive disorders of pregnancy (HDP) (5 trials) [44, 46, 47, 49, 50], FGR (7 trials) [52, 53, 54, 57, 58, 60, 61], HDP and FGR (1 trial) [59]; or women presenting in preterm labour (1 trial) [51]. One trial recruited primigravidas not at high risk [62]. The L‐citrulline trial was conducted in the UK and included 36 pregnant women with chronic hypertension [45]. Fourteen RCTs were considered prevention trials [44, 45, 46, 47, 49, 50, 51, 52, 53, 54, 57, 58, 60, 61, 62], five treatment trials [38, 39, 48, 55, 56, 59], and one included both women with pre‐eclampsia (treatment) and without pre‐eclampsia (prevention) [47].

Four L‐arginine trials commenced supplementation in the second trimester (14–20 weeks) [46, 49, 50, 62], six started from the second to third trimester (24–36 weeks) [38, 39, 44, 47, 51, 57, 60], and two started in the third trimester (28–40 weeks) [54, 55]; seven trials did not define when supplementation started [48, 52, 53, 56, 58, 59, 61]. Supplementation lasted: until birth or up to 3 days postpartum in five trials [38, 39, 44, 48, 56, 57]; for 1 week or less in two trials [55, 58]; 2–4 weeks in five trials [47, 53, 54, 61]; and 10–12 weeks in two trials [50, 62]; five trials did not state the duration of supplementation [46, 49, 52, 59, 60]. The L‐citrulline trial supplemented for 8 weeks commencing at 12–16 weeks gestation [45].

The dose and mode of administration of L‐arginine differed across trials. Fourteen trials administered L‐arginine orally. Eight used 3 g/day tablets [38, 39, 46, 51, 53, 54, 56, 60, 61] (one involved 1 g three times daily [56]), two used 4 g/day tablets [49, 50], one used 4 g tablets three times daily [55], one used 6.6 g/day as dietary bars [43, 44], one used 1 g daily [62], and one used 14 g/day (90 mL) oral syrup in two equal doses [57]. Three trials administered L‐arginine intravenously—two used 20 g/day [58, 59] and one used 15 g/day [52]. One trial used 20 g/day intravenous fluid for 5 days followed by 4 g/day oral tablets for 2 weeks [47], and one trial used either 3.5 g oral tablets every 6 h or 10 g intravenous fluid every 8 h when L‐arginine could not be taken orally [48]. The comparison groups received a placebo in 11 trials [38, 39, 46, 47, 48, 50, 54, 55, 56, 57, 60], routine or conventional treatment in four [52, 58, 59, 61], antioxidant vitamins in one [44], prenatal supplements in one [62] and no treatment or ‘observation only’ in two trials [49, 53]. L‐citrulline was administered at a dose of 3 g (30 mL oral syrup) twice daily, and the comparison group received a placebo [45] (Table S4).

Nine RCTs were judged to have a high risk of bias and 13 had some concerns (Figure S2). All RCTs had ‘some concerns’ of bias in the selection of reported results due either to the unavailability of a pre‐specified analysis plan or additional post hoc analysis. None of the included RCTs had been retracted or flagged for integrity concerns.

### 3.1 Findings From Randomised Controlled Trials of L‐Arginine for Pre‐Eclampsia Prevention

#### 3.1.1 Primary Outcomes

**FIGURE 2:**
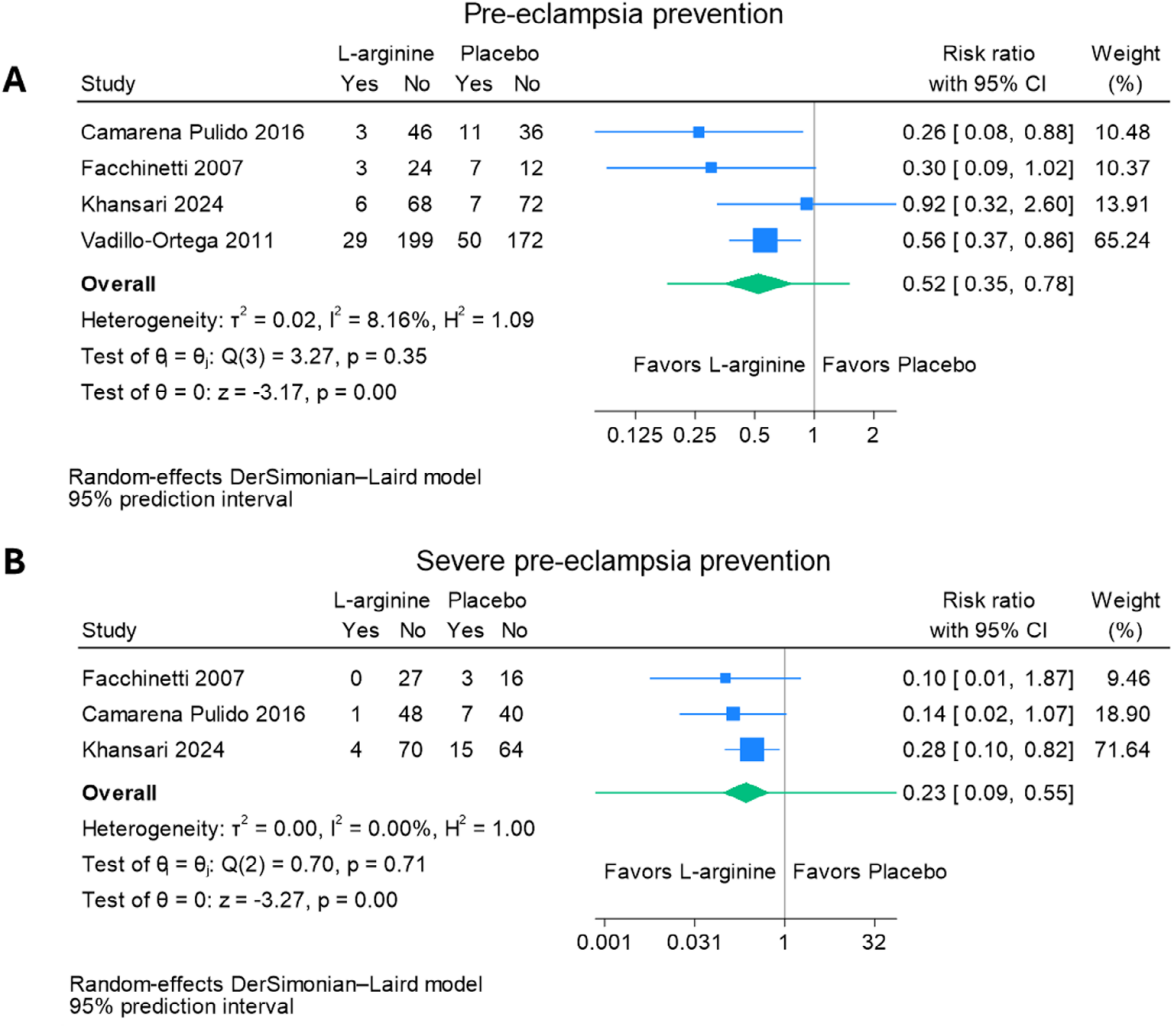
Forest plots of primary outcomes: (A) pre‐eclampsia prevention and (B) severe pre‐eclampsia prevention.

Data from four trials (involving 745 women at high risk of pre‐eclampsia) showed that L‐arginine may reduce the risk of pre‐eclampsia (relative risk [RR], 0.52; 95% CI, 0.35, 0.78; low certainty evidence) [43, 44, 46, 47] and severe pre‐eclampsia in women at risk of pre‐eclampsia (RR 0.23; 95% CI, 0.09, 0.55; three trials, 295 women; low certainty evidence) [46, 47] (Figure 2, Appendix S3).

#### 3.1.2 Secondary Maternal Outcomes

**FIGURE 3:**
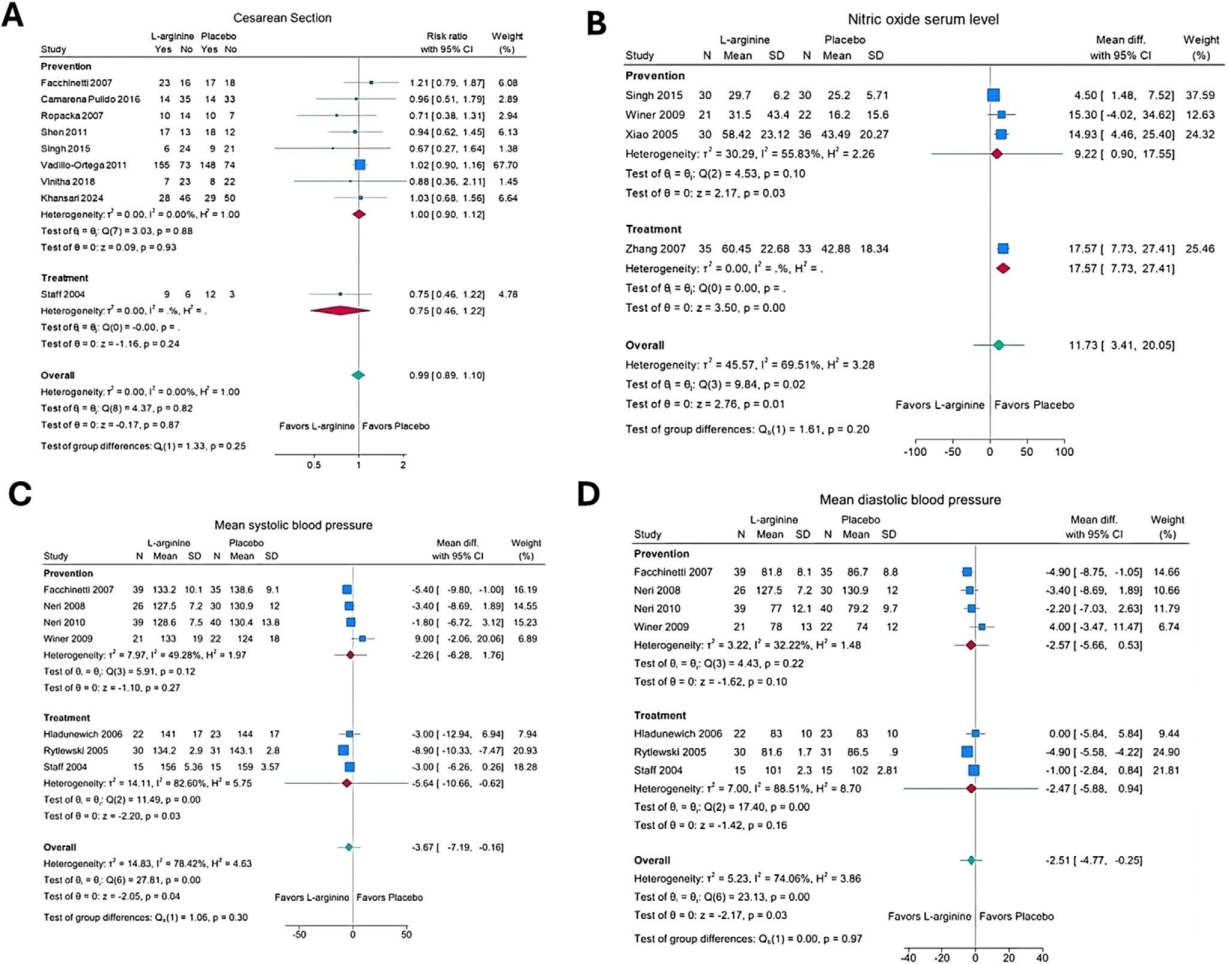
Forest plots of secondary maternal outcomes: (A) caesarean section, (B) nitric oxide serum level, (C) mean systolic blood pressure and (D) mean diastolic blood pressure.

L‐arginine may have no effect on the risk of caesarean section in women at risk of pre‐eclampsia (RR 1.00; 95% CI, 0.90, 1.12; eight trials, 994 women; low certainty evidence). L‐arginine may increase serum levels of nitric oxide compared to placebo (mean difference [MD] 9.22 μmol/L; 95% CI 0.90, 17.55; three trials; 169 women; low certainty evidence) in women at risk of pre‐eclampsia [54, 57, 58]. We found no association of L‐arginine with mean systolic or diastolic BP, or the risk of adverse effects, from very low‐certainty evidence in women at risk of pre‐eclampsia (Figure 3, Figure S3, Appendix S3).

#### 3.1.3 Secondary Fetal/Neonatal Outcomes

**FIGURE 4:**
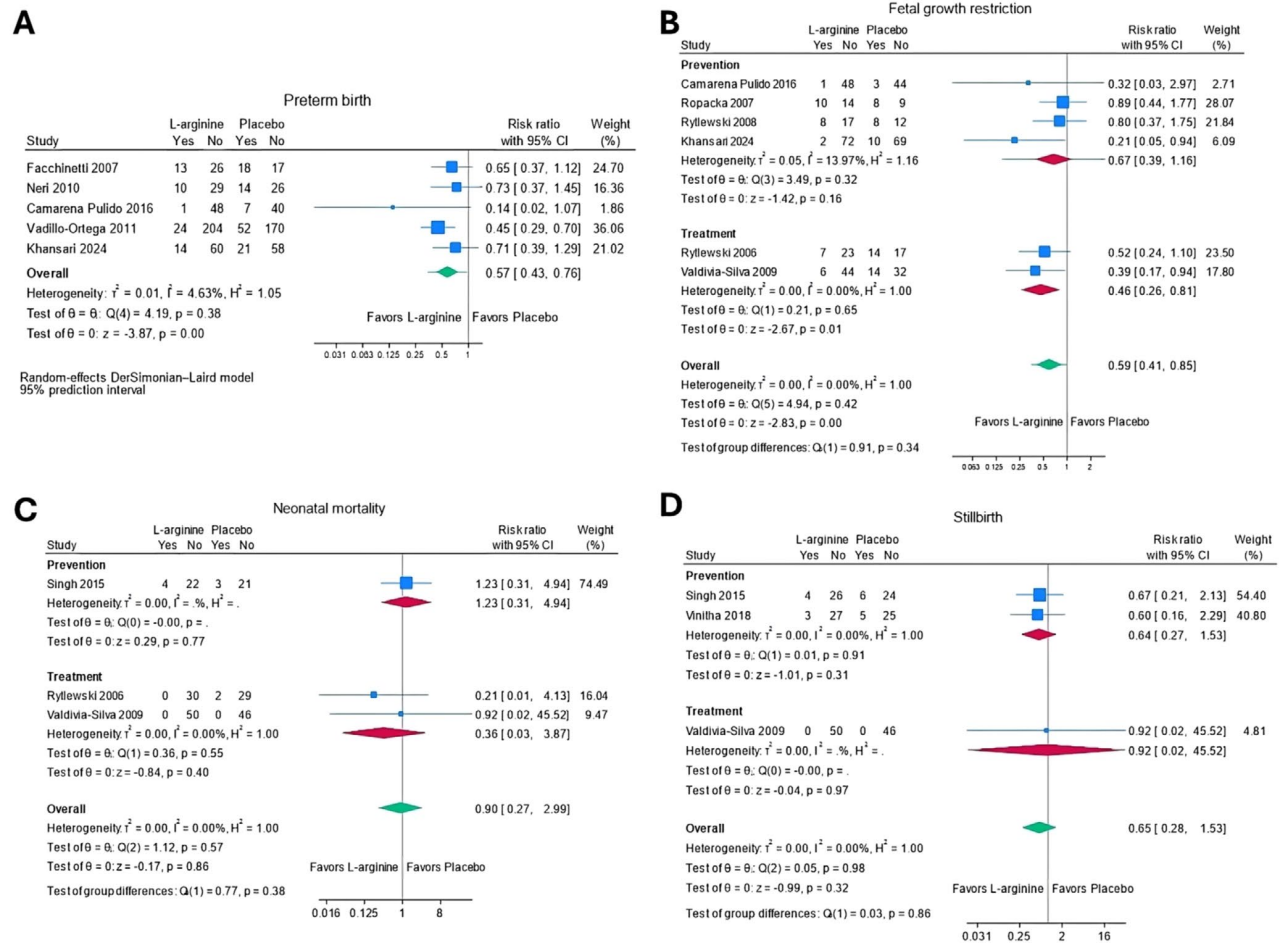
Forest plots of secondary fetal/neonatal outcomes: (A) preterm birth, (B) fetal growth restriction, (C) neonatal mortality and (D) stillbirth.

L‐arginine may decrease the risk of preterm birth < 37 weeks gestation (RR 0.57; 95% CI 0.43, 0.76; five trials; 852 women; low certainty evidence) in women at risk of pre‐eclampsia [43, 44, 46, 47, 50, 62]. We are uncertain of the effect of L‐arginine on small‐for‐gestational age (SGA) infants in women at risk of pre‐eclampsia (RR 0.39; 95% CI 0.27, 0.56; two trials; 168 women; very low certainty evidence) [52, 53]. L‐arginine may increase birth weight (MD 150.13 g; 95% CI 56.03, 244.24; 14 trials; 1391 women; low certainty evidence) [44, 46, 47, 49, 50, 51, 52, 53, 54, 57, 58, 60, 61, 62] and gestational age at birth (MD 0.50 weeks; 95% CI 0.15, 0.84; 12 trials; 1235 women; low certainty evidence) in women at risk of pre‐eclampsia [43, 44, 47, 49, 50, 51, 53, 54, 57, 58, 60, 61, 62]. We found no association between L‐arginine and the risk of admission to the neonatal intensive care unit (NICU), FGR, neonatal mortality, stillbirth, low birth weight < 2500 g, neonatal hypoglycaemia, respiratory distress syndrome and neonatal infections from very low‐certainty evidence in women at risk of pre‐eclampsia (Figure 4, Figure S4, Appendix S3).

### 3.2 Findings From Randomised Controlled Trials of L‐Arginine for Pre‐Eclampsia Treatment

#### 3.2.1 Primary Outcome

Four trials (reported in five studies) included women with diagnosed pre‐eclampsia, but none reported on the primary treatment outcomes (progression to severe pre‐eclampsia, eclampsia and/or HELLP syndrome) [38, 39, 48, 55, 56].

#### 3.2.2 Secondary Maternal Outcomes

L‐arginine may reduce mean systolic blood pressure in women with pre‐eclampsia, but the evidence is very uncertain (MD −5.64; 95% CI, −10.66, −0.62; three trials, 136 women; very low certainty evidence) [38, 48, 55]. L‐arginine may increase serum levels of nitric oxide compared with placebo (mean difference [MD] 17.55 μmol/L; 95% CI 7.73, 27.41; one trial; 68 women; low certainty evidence) [59]. We found no association of L‐arginine with caesarean section or mean diastolic BP from very low certainty evidence in women with pre‐eclampsia (Figure 3, Appendix S4).

#### 3.2.3 Secondary Fetal/Neonatal Outcomes

L‐arginine may increase birth weight (MD 215.83 g; 95% CI 87.33, 344.34; three trials; 159 women; low certainty evidence) [39, 55, 59]. L‐arginine may decrease the risk of FGR in women with pre‐eclampsia (RR 0.46; 95% CI, 0.26, 0.81; two trials, 157 women; low certainty evidence) [39, 56]. We found no association of L‐arginine with admission to NICU, mean gestational age at birth, neonatal mortality or stillbirth from low‐ or very low‐certainty evidence in women with pre‐eclampsia (Figure 4, Figure S4, Appendix S4).

### 3.3 Findings From Randomised Controlled Trials of L‐Citrulline

L‐citrulline did not have any effect on the risk of pre‐eclampsia (RR 0.83; 95% CI, 0.24, 2.92; 1 trial; 36 women) or systolic or diastolic BP in women with chronic hypertension. L‐citrulline significantly increased maternal plasma levels of L‐citrulline and L‐arginine [45].

Findings from subgroup analyses are reported in Figure S5, and findings from non‐RCTs are reported in Figures S6 and S7.

## 4 Discussion

### 4.1 Main Findings

This is the first systematic review to evaluate the evidence on L‐arginine and L‐citrulline for the prevention or treatment of pre‐eclampsia, separately. This distinction is critical for informing clinical practice and policy, as different stages of the aetiology are targeted by prevention or treatment trials. We found low‐certainty evidence that L‐arginine in pregnancy decreases the risk of pre‐eclampsia and severe pre‐eclampsia among women at risk of pre‐eclampsia. The evidence is very uncertain about the effect of L‐arginine on mean systolic BP, and L‐arginine may decrease the risk of FGR in treatment trials. L‐arginine may also decrease the risk of preterm birth and SGA and increase nitric oxide serum levels in prevention trials. We are uncertain of the effects of L‐arginine on other secondary outcomes.

### 4.2 Strengths and Limitations

This review is the first to explore the effects of L‐arginine and L‐citrulline for prevention separately from the treatment of pre‐eclampsia, which has significant implications for the optimal timing of supplementation. We included data from RCTs and assessed all trials and their authors for research integrity to ensure that the review was devoid of retracted or problematic trials. We also included only studies in which the treatment and control groups were from the same population to avoid bias. The main limitation of this meta‐analysis is the absence of large, prospectively registered trials reporting on our primary outcomes. Most data came from participants at risk of pre‐eclampsia, other hypertensive disorders of pregnancy, or with FGR. Although, for some secondary outcomes, we performed subgroup analysis by risk categories of the included population, we could not do this for the primary outcomes. Also, we could not perform subgroup analysis by gestational age at the start of supplementation because of the few trials and variation between them. Furthermore, all studies had some concerns regarding bias, but this was accounted for by downgrading the certainty of evidence in the GRADE assessment. In addition, the imprecision of the effect estimates, due to the small sample sizes, lowered the certainty of evidence.

### 4.3 Interpretation

Consistent with previous reviews, we found that L‐arginine may be promising for preventing pre‐eclampsia [26, 27]. However, this data should be interpreted with caution, as the certainty of the evidence is low, requiring further research. Our review defined trials including women without a diagnosis of pre‐eclampsia as prevention trials; however, only four trials specifically examined the efficacy of L‐arginine to prevent pre‐eclampsia in women at increased risk. These trials included women who were nulliparous, had a previous history of pre‐eclampsia, had pre‐eclampsia in a first‐degree relative, had chronic hypertension, had gestational hypertension without proteinuria or had a body mass index ≥ 30 [63]. None of the trials used currently recommended screening tools to identify women at risk of pre‐eclampsia, such as the Fetal Monitoring Foundation (FMF) algorithm [64]. The optimal timing for initiating supplementation also remains unclear. Only two trials started supplementation before 20 weeks [49, 50], and seven did not report the time of initiation of supplementation. This is important given that the currently recommended preventive agent, aspirin, is known to be effective for pre‐eclampsia prevention only if started before 20 weeks gestation [4].

Previous systematic reviews of the effects of L‐arginine on pre‐eclampsia have not explored its effects on treating women with established pre‐eclampsia [24, 27, 65, 66]. Our review demonstrates the potential benefit of L‐arginine for reducing mean systolic BP (a key indicator of pre‐eclampsia) in women diagnosed with pre‐eclampsia. However, only three trials contributed to this. More and larger trials are needed, which also report on other treatment outcomes like progression to severe pre‐eclampsia and eclampsia. New treatments for pre‐eclampsia would be of great benefit to women globally, particularly in settings where risk screening and preventive agents are poorly implemented.

L‐arginine may be beneficial in low‐middle‐income countries (LMICs) that are malaria‐endemic, given that malaria in pregnancy increases the risk of pre‐eclampsia [67]. Malaria reduces the bioavailability of L‐arginine and nitric oxide and worsens disease severity [20]. It is noteworthy, however, that only two RCTs were conducted in an LMIC (India). This is of concern, considering that pre‐eclampsia and eclampsia account for up to 10%–15% of direct maternal mortality, and in LMICs, the maternal mortality rate was 430/100 000 live births in 2020 compared to 12/100 000 live births in high‐income countries [68, 69]. Prenatal supplements are widely used by pregnant women in many settings globally [70, 71]. However, L‐arginine is not currently included in standard prenatal supplements [72]. If proven effective for pre‐eclampsia prevention, L‐arginine could be added to prenatal supplements due to its stability and considered for addition to the United Nations International Multiple Micronutrient Antenatal Preparation Multiple Micronutrient Supplements.

Further research is needed to establish the optimal time to start L‐arginine for pre‐eclampsia prevention and the population that would most benefit from supplementation. An individual participant data meta‐analysis may be able to answer this question. For example, the largest trial (672 women) commenced supplementation from 14 to 32 weeks but did not report subgroup findings by time of commencing supplementation [44]. The effects of co‐administration of L‐arginine with aspirin or calcium (known prevention therapies) are unknown and should be explored in future trials. Previous studies have suggested differences in the pathophysiology of early‐onset and late‐onset pre‐eclampsia [73, 74, 75], which may impact the effectiveness of L‐arginine for prevention or treatment. The included trials did not define the sub‐classification of pre‐eclampsia in their inclusion criteria or outcomes. Future trials that define the type of pre‐eclampsia experienced would provide insight into the optimal dose and gestational age to commence L‐arginine to maximise its benefit. Furthermore, L‐citrulline has been shown to increase the bioavailability of L‐arginine and augment nitric oxide production, thereby exerting a greater benefit than supplementation with L‐arginine alone [20, 23, 76]. However, there was only one small trial on L‐citrulline during pregnancy which did not demonstrate any effect on BP despite evidence of such effect in trials of non‐pregnant individuals. The trial authors highlighted the need for future studies to investigate the pharmacodynamic and pharmacokinetic profile of L‐citrulline in pregnant women as this may differ from other adult populations [45].

## 5 Conclusion

L‐arginine is promising for pre‐eclampsia prevention, and we are uncertain of its effect as a treatment for women with established pre‐eclampsia. L‐arginine could constitute an important addition to the management protocol of women at risk of pre‐eclampsia, but further research is needed to establish the population that would benefit, the optimal dose, time of initiation and duration of supplementation. An individual participant data meta‐analysis may provide definitive answers to these questions. Future L‐arginine trials should use standardised pre‐eclampsia screening tools, define the type of pre‐eclampsia experienced and investigate co‐administration with aspirin or calcium.

## Author Contributions

M.M., A.R.A.M., A.M.G. and J.P.V. conceived the study. M.M., A.R.A.M. and J.P.V. designed the protocol. M.M. and A.R.A.M. developed the search strategy. M.M., A.R.A.M., J.C. and K.M. selected the studies and extracted relevant information. M.M., A.R.A.M. and J.P.V. synthesised the data. M.M. wrote the first draft of the paper. All authors contributed to the interpretation of the findings and critically revised successive drafts of the paper.

## Conflicts of Interest

The authors declare no conflicts of interest.

## Supporting information


Appendix S1.

Appendix S2.

Appendix S3.

Appendix S4.

Data S1.

## Data Availability

The data that support the findings of this study are available within the article and its Supporting Information.
