# Supplementary material for: L‐Arginine and L‐Citrulline for Prevention and Treatment of Pre‐Eclampsia: A Systematic Review and Meta‐Analysis
Source: BJOG. 2025 Jan 12;132(6):698–708. doi: 10.1111/1471-0528.18070 (PMC11969923; doi:10.1111/1471-0528.18070)
Supplement: Supplementary file 1 — Appendix S1. [file BJO-132-698-s003.pdf]

Appendix S1 – Search strategy

Ovid MEDLINE(R) ALL <1946 to February 07, 2024>

1 Arginine/

2 arginine.mp.

3 citrulline/

4 citrul?in?.mp.

5 1 or 2 or 3 or 4

6 pregnan*.mp.

7 (expectant adj (mother* or wom#n or girl* or female*)).mp.

8 6 or 7

9 5 and 8

10 exp animals/ not humans.sh.

11 9 not 10

Embase Classic+Embase <1947 to 2024 February 07>

1 Arginine/

2 arginine.mp.

3 citrulline/

4 citrul?in?.mp.

5 1 or 2 or 3 or 4

6 pregnan*.mp.

7 (expectant adj (mother* or wom#n or girl* or female*)).mp.

8 6 or 7

9 5 and 8

10 (exp animal/ or nonhuman/ or animal.hw. or invertebrates/) not human.sh.

11 9 not 10

EBM Reviews - Cochrane Central Register of Controlled Trials <07 February 2024>

1 Arginine/

2 arginine.mp.

3 citrulline/

4 citrul?in?.mp.

5 1 or 2 or 3 or 4

6 pregnan*.mp.

7 (expectant adj (mother* or wom#n or girl* or female*)).mp.

8 6 or 7

9 5 and 8

10 exp animals/ not humans.sh.

11 9 not 10

Global index medicus 07/02/2024

tw:((tw:((tw:((arginine OR citrulline))) AND (tw:((pregnancy OR pregnant))))))

EBSCOhost Research Databases - CINAHL Plus 7 February 2024

February 07, 2024	**Query**	**Limiters/Expanders**
S7	S5 NOT S6	Search modes -Boolean/Phrase
S6	(MH "Animals+") NOT(MH "Human")	Search modes -Boolean/Phrase
S5	S1 AND S4	Search modes -Boolean/Phrase
S4	S2 OR S3	Search modes -Boolean/Phrase
S3	arginine or l-arginine	Search modes -Boolean/Phrase
S2	citruline or citrullin orcitrulin or citrulline or l-citrulline or l citrulline	Search modes -Boolean/Phrase
S1	pregnant women orpregnant woman orpregnancy or pregnant orexpecting mother	Search modes -Boolean/Phrase
