# Supplementary material for: L‐Arginine and L‐Citrulline for Prevention and Treatment of Pre‐Eclampsia: A Systematic Review and Meta‐Analysis
Source: BJOG. 2025 Jan 12;132(6):698–708. doi: 10.1111/1471-0528.18070 (PMC11969923; doi:10.1111/1471-0528.18070)
Supplement: Supplementary file 2 — Appendix S2. [file BJO-132-698-s005.pdf]

Appendix S2 – Primary and Secondary Outcomes

Primary outcomes

- Incidence of pre-eclampsia (prevention outcome)
- Progression to severe pre-eclampsia, eclampsia and/or HELLP syndrome (treatment outcome)

Secondary outcomes

Maternal

- Preterm birth (<37 weeks' gestation)
- Biomarkers of pre-eclampsia (sFlt-1, placental growth factor (PlGF), soluble endoglin PAPP-A, Endothelin-1 etc)
- Maternal L-arginine plasma level (post-intervention)
- Maternal plasma levels of nitric oxide (post-intervention)
- Maternal death (as defined by authors)
- Miscarriage (pregnancy loss before 20 weeks' gestation)
- Maternal infectious morbidity outcomes: maternal sepsis, bacterial vaginosis, chorioamnionitis, postpartum endometritis, postnatal fever
- Maternal hypertension morbidity outcomes: pregnancy-induced hypertension, pre-eclampsia, eclampsia
- Admission to ICU
- Adverse effects of intervention (including mild effects: diarrhea, nausea/vomiting, abdominal pain, bloating, and severe effects: respiratory distress syndrome, inflammation of the airways, cardiac arrest, myocardial infarction, or renal failure)
- Maternal well-being or satisfaction (as defined by study authors)
- Any composite measure of the aforementioned maternal outcomes

Fetus/neonate:

- Mortality:
    - Perinatal mortality (composite of fetal death and early neonatal death, or as defined by trial authors)
    - Neonatal mortality (death of liveborn neonate within 28 days of life, or as defined by trial authors)

    - Fetal death / stillbirth (as reported by trial authors)
    - Infant or childhood death
- Severe newborn morbidity i.e., an illness in the neonatal period that is associated with a high risk of death or severe long-term disability among survivors, e.g.,
    - moderate/severe respiratory distress syndrome (RDS)
    - severe intraventricular haemorrhage (grade III/IV)
    - severe neonatal infection
    - necrotising enterocolitis
    - chronic lung disease/bronchopulmonary dysplasia
    - periventricular leukomalacia
    - retinopathy of prematurity
    - patent ductus arteriosus
    - admission to the Neonatal Intensive Care Unit (NICU)
- Respiratory distress syndrome (as defined by authors)
- Neonatal hypoglycaemia
- Small for gestational age (as defined by authors)
- Fetal growth restriction (as defined by authors)
- Low birthweight (<2500g)
- Birthweight (as defined by authors)
- Preterm birth: <28 weeks; <32 weeks; <34 weeks; <37 weeks
- Gestational age at birth
- Harm to offspring from intervention (birth defects including congenital heart defects, congenital diaphragmatic hernia)
- Long-term morbidity (i.e., an illness occurring after the neonatal period that is associated with physical or behavioural impairment among survivors, e.g., cerebral palsy, developmental delay, intellectual, hearing, or visual impairment)
- Any composite measure of the aforementioned neonatal outcomes
