# Supplementary material for: L‐Arginine and L‐Citrulline for Prevention and Treatment of Pre‐Eclampsia: A Systematic Review and Meta‐Analysis
Source: BJOG. 2025 Jan 12;132(6):698–708. doi: 10.1111/1471-0528.18070 (PMC11969923; doi:10.1111/1471-0528.18070)
Supplement: Supplementary file 3 — Appendix S3. [file BJO-132-698-s001.pdf]

Primary outcomes – prevention trials

L-arginine compared to placebo or no treatment for the prevention of preeclampsia in pregnant women

Patient or population: the prevention of preeclampsia in pregnant women

Intervention: L-arginine

Comparison: placebo or no treatment

Outcomes	№ of participants (studies) Follow-up	Certainty of the evidence (GRADE)	Relative effect (95% CI)	Anticipated absolute effects	
				Risk with placebo or no treatment	Risk difference with L-arginine
Preeclampsia - prevention trials	745 (4 RCTs)	⊕⊕◯◯ Low[a,b,c,d]	**RR 0.52** (0.35 to 0.78)	204 per 1,000	**98 fewer per 1,000** (133 fewer to 45 fewer)
Severe preeclampsia - prevention trials	295 (3 RCTs)	⊕⊕◯◯ Low[c,d,e,f]	**RR 0.23** (0.09 to 0.55)	172 per 1,000	**133 fewer per 1,000** (157 fewer to 78 fewer)

*__The risk in the intervention group__ (and its 95% confidence interval) is based on the assumed risk in the comparison group and the **relative effect** of the intervention (and its 95% CI).

CI: confidence interval; **MD:** mean difference; **RR:** risk ratio

GRADE Working Group grades of evidence
High certainty: we are very confident that the true effect lies close to that of the estimate of the effect.
Moderate certainty: we are moderately confident in the effect estimate: the true effect is likely to be close to the estimate of the effect, but there is a possibility that it is substantially different.
Low certainty: our confidence in the effect estimate is limited: the true effect may be substantially different from the estimate of the effect.
Very low certainty: we have very little confidence in the effect estimate: the true effect is likely to be substantially different from the estimate of effect.

Explanations

a. Only one study has a high risk of bias.

b. 1 out of 4 studies have a high risk of bias

c. Few events

d. There are not enough studies to test for publication bias

e. 2 out of 4 studies have a high risk of bias

f. Wide confidence interval

Secondary maternal outcomes – prevention trials

L-arginine compared to placebo or no treatment for the prevention of preeclampsia in pregnant women

Patient or population: the prevention of preeclampsia in pregnant women

Intervention: L-arginine

Comparison: placebo or no treatment

Outcomes	№ of participants (studies) Follow-up	Certainty of the evidence (GRADE)	Relative effect (95% CI)	Anticipated absolute effects	
				Risk with placebo or no treatment	Risk difference with L-arginine
Cesarean section - prevention trials	994 (8 RCTs)	⊕⊕◯◯ Low[c,d,e]	**RR 1.00** (0.90 to 1.12)	516 per 1,000	**0 fewer per 1,000** (52 fewer to 62 more)
Mean diastolic blood pressure - prevention trials	252 (4 RCTs)	⊕◯◯◯ Very low[c,d,f]	-		MD **2.57 mmHg lower** (5.66 lower to 0.53 higher)
Mean systolic blood pressure - prevention trials	252 (4 RCTs)	⊕◯◯◯ Very low[c,d,f]	-		MD **2.26 mmHg lower** (6.28 lower to 1.76 higher)
Adverse effects - prevention trials	328 (3 RCTs)	⊕◯◯◯ Very low[b,c,d,g]	**RR 2.63** (0.95 to 7.32)	56 per 1,000	**91 more per 1,000** (3 fewer to 353 more)

Secondary maternal outcomes – prevention trials

L-arginine compared to placebo or no treatment for the prevention of preeclampsia in pregnant women

Patient or population: the prevention of preeclampsia in pregnant women

Intervention: L-arginine

Comparison: placebo or no treatment

Outcomes	№ of participants (studies) Follow-up	Certainty of the evidence (GRADE)	Relative effect (95% CI)	Anticipated absolute effects	
				Risk with placebo or no treatment	Risk difference with L-arginine
Nitric oxide serum level - prevention trials	169 (3 RCTs)	⊕⊕◯◯ Low[c,h]	-		MD **9.22 µmol/L higher** (0.9 higher to 17.55 higher)

***The risk in the intervention group** (and its 95% confidence interval) is based on the assumed risk in the comparison group and the **relative effect** of the intervention (and its 95% CI).
[#]The absolute risk difference should be interpreted with caution due to the small number of trials and few or no events in either group
CI: confidence interval; **MD:** mean difference; **RR:** risk ratio

GRADE Working Group grades of evidence
High certainty: we are very confident that the true effect lies close to that of the estimate of the effect.
Moderate certainty: we are moderately confident in the effect estimate: the true effect is likely to be close to the estimate of the effect, but there is a possibility that it is substantially different.
Low certainty: our confidence in the effect estimate is limited: the true effect may be substantially different from the estimate of the effect.
Very low certainty: we have very little confidence in the effect estimate: the true effect is likely to be substantially different from the estimate of effect.

Explanations

a. 1 out of 4 studies have a high risk of bias

b. Few events

c. There are not enough studies to test for publication bias

d. Wide confidence intervals and includes no effect

e. 2 out of 7 studies have a high risk of bias

f. Findings of 1 trial not consistent with the others

g. 2 out of 4 studies have a high risk of bias

h. Wide confidence interval

Secondary neonatal outcomes – prevention trials

L-arginine compared to placebo or no treatment for the prevention of preeclampsia in pregnant women

Patient or population: the prevention of preeclampsia in pregnant women

Intervention: L-arginine

Comparison: placebo or no treatment

Outcomes	№ of participants (studies) Follow-up	Certainty of the evidence (GRADE)	Relative effect (95% CI)	Anticipated absolute effects	
				Risk with placebo or no treatment	Risk difference with L-arginine
Preterm birth - prevention trials	852 (5 RCTs)	⊕⊕◯◯ Low[a,b,c]	**RR 0.57** (0.43 to 0.76)	265 per 1,000	**114 fewer per 1,000** (151 fewer to 64 fewer)
Mean birth weight - prevention trials	1391 (14 RCTs)	⊕⊕◯◯ Low[d,e]	-		MD **150.13 g higher** (56.03 higher to 244.24 higher)
Neonatal mortality - prevention trials	50 (1 RCT)	⊕◯◯◯ Very low[b,c,f]	**RR 1.23** (0.31 to 4.94)	125 per 1,000[#]	**29 more per 1,000** (86 fewer to 493 more)
Neonatal intensive unit admissions - prevention trials	315 (5 RCTs)	⊕◯◯◯ Very low[b,c,f,g]	**RR 0.56** (0.31 to 1.04)	248 per 1,000	**109 fewer per 1,000** (171 fewer to 10 more)
Stillbirth - prevention trials	120 (2 RCTs)	⊕◯◯◯ Very low[b,c,f,h]	**RR 0.64** (0.27 to 1.53)	183 per 1,000[#]	**66 fewer per 1,000** (134 fewer to 97 more)
Mean gestational age at birth - prevention trials	1235 (12 RCTs)	⊕⊕◯◯ Low[i,j]	-		MD **0.41 weeks higher** (0.06 higher to 0.76 higher)

Secondary neonatal outcomes – prevention trials

L-arginine compared to placebo or no treatment for the prevention of preeclampsia in pregnant women

Patient or population: the prevention of preeclampsia in pregnant women

Intervention: L-arginine

Comparison: placebo or no treatment

Outcomes	№ of participants (studies) Follow-up	Certainty of the evidence (GRADE)	Relative effect (95% CI)	Anticipated absolute effects	
				Risk with placebo or no treatment	Risk difference with L-arginine
Fetal growth restriction - prevention trials	335 (4 RCTs)	⊕◯◯◯ Very low[b,c,f,k]	**RR 0.67** (0.39 to 1.16)	178 per 1,000	**59 fewer per 1,000** (109 fewer to 28 more)
Low birth weight - prevention trials	153 (2 RCTs)	⊕⊕◯◯ Low[b,c,f]	**RR 0.66** (0.40 to 1.09)	347 per 1,000[#]	**118 fewer per 1,000** (208 fewer to 31 more)
Neonatal hypoglycemia - prevention trials	102 (2 RCTs)	⊕◯◯◯ Very low[b,c,f,h]	**RR 0.52** (0.27 to 1.02)	367 per 1,000[#]	**176 fewer per 1,000** (268 fewer to 7 more)
Respiratory distress syndrome - prevention trials	143 (3 RCTs)	⊕◯◯◯ Very low[b,c,f,k,l]	**RR 0.64** (0.36 to 1.14)	591 per 1,000	**213 fewer per 1,000** (378 fewer to 83 more)
Small for gestational age - prevention trials	168 (2 RCTs)	⊕◯◯◯ Very low[b,c,h]	**RR 0.39** (0.27 to 0.56)	617 per 1,000[#]	**376 fewer per 1,000** (450 fewer to 271 fewer)
Neonatal infections - prevention trials	41 (1 RCT)	⊕◯◯◯ Very low[b,c,f]	**RR 0.71** (0.16 to 3.10)	176 per 1,000[#]	**51 fewer per 1,000** (148 fewer to 371 more)

Secondary neonatal outcomes – prevention trials

L-arginine compared to placebo or no treatment for the prevention of preeclampsia in pregnant women

Patient or population: the prevention of preeclampsia in pregnant women

Intervention: L-arginine

Comparison: placebo or no treatment

Outcomes	№ of participants (studies) Follow-up	Certainty of the evidence (GRADE)	Relative effect (95% CI)	Anticipated absolute effects	
				Risk with placebo or no treatment	Risk difference with L-arginine

***The risk in the intervention group** (and its 95% confidence interval) is based on the assumed risk in the comparison group and the **relative effect** of the intervention (and its 95% CI).
[#]The absolute risk difference should be interpreted with caution due to the small number of trials and few or no events in either group
CI: confidence interval; **MD:** mean difference; **RR:** risk ratio

GRADE Working Group grades of evidence
High certainty: we are very confident that the true effect lies close to that of the estimate of the effect.
Moderate certainty: we are moderately confident in the effect estimate: the true effect is likely to be close to the estimate of the effect, but there is a possibility that it is substantially different.
Low certainty: our confidence in the effect estimate is limited: the true effect may be substantially different from the estimate of the effect.
Very low certainty: we have very little confidence in the effect estimate: the true effect is likely to be substantially different from the estimate of effect.

Explanations

a. Only one study has a high risk of bias.

b. Few events

c. There are not enough studies to test for publication bias

d. 5 out of 14 studies have high risk of bias

e. Some confidence intervals do not overlap. Considerable heterogeneity (I2 = 85.63%)

f. Wide confidence intervals and includes no effect

g. 1 out of 5 studies have a high risk of bias

h. 2 out of 3 studies have a high risk of bias

i. 5 out of 11 studies have high risk of bias

j. Publication bias suspected, P for egger's test = 0.0162

k. 2 out of 4 studies have a high risk of bias

l. Substantial heterogeneity based on I2
