# Supplementary material for: L‐Arginine and L‐Citrulline for Prevention and Treatment of Pre‐Eclampsia: A Systematic Review and Meta‐Analysis
Source: BJOG. 2025 Jan 12;132(6):698–708. doi: 10.1111/1471-0528.18070 (PMC11969923; doi:10.1111/1471-0528.18070)
Supplement: Supplementary file 4 — Appendix S4. [file BJO-132-698-s004.pdf]

Secondary maternal outcomes – treatment trials

L-arginine compared to placebo or no treatment for the treatment of preeclampsia in pregnant women

Patient or population: the treatment of preeclampsia in pregnant women

Intervention: L-arginine

Comparison: placebo or no treatment

Outcomes	№ of participants (studies) Follow-up	Certainty of the evidence (GRADE)	Relative effect (95% CI)	Anticipated absolute effects	
				Risk with placebo or no treatment	Risk difference with L-arginine
Cesarean section - treatment trials	30 (1 RCT)	⊕◯◯◯ Very low[a,b,c]	**RR 0.75** (0.46 to 1.22)	800 per 1,000[#]	**200 fewer per 1,000** (432 fewer to 176 more)
Mean systolic blood pressure - treatment trials	136 (3 RCTs)	⊕◯◯◯ Very low[b,c,d,e]	-		MD **5.64 mmHg lower** (10.66 lower to 0.62 lower)
Mean diastolic blood pressure - treatment trials	136 (3 RCTs)	⊕◯◯◯ Very low[b,c,d,f]	-		MD **2.47 mmHg lower** (5.88 lower to 0.94 higher)

***The risk in the intervention group** (and its 95% confidence interval) is based on the assumed risk in the comparison group and the **relative effect** of the intervention (and its 95% CI).
[#]The absolute risk difference should be interpreted with caution due to the small number of trials and few or no events in either group
CI: confidence interval; **MD:** mean difference; **RR:** risk ratio

GRADE Working Group grades of evidence
High certainty: we are very confident that the true effect lies close to that of the estimate of the effect.
Moderate certainty: we are moderately confident in the effect estimate: the true effect is likely to be close to the estimate of the effect, but there is a possibility that it is substantially different.
Low certainty: our confidence in the effect estimate is limited: the true effect may be substantially different from the estimate of the effect.
Very low certainty: we have very little confidence in the effect estimate: the true effect is likely to be substantially different from the estimate of effect.

Explanations

a. Few events

b. There are not enough studies to test for publication bias

c. Wide confidence intervals and includes no effect

d. 1 out of 3 studies have a high risk of bias

e. Considerable heterogeneity I2 = 82.6%

f. Considerable heterogeneity I2 = 88.51%

Secondary neonatal outcomes – treatment trials

L-arginine compared to placebo or no treatment for the treatment of preeclampsia in pregnant women

Patient or population: the treatment of preeclampsia in pregnant women

Intervention: L-arginine

Comparison: placebo or no treatment

Outcomes	№ of participants (studies) Follow-up	Certainty of the evidence (GRADE)	Relative effect (95% CI)	Anticipated absolute effects	
				Risk with placebo or no treatment	Risk difference with L-arginine
Neonatal intensive care unit admissions - treatment trials	30 (1 RCT)	⨁◯◯◯ Very low[a,b,d]	**RR 1.00** (0.36 to 2.75)	333 per 1,000[#]	**0 fewer per 1,000** (213 fewer to 583 more)
Fetal growth restriction - treatment trials	157 (2 RCTs)	⨁⨁◯◯ Low[a,b,c,g]	**RR 0.46** (0.26 to 0.81)	364 per 1,000[#]	**196 fewer per 1,000** (269 fewer to 69 fewer)
Mean birth weight - treatment trials	159 (3 RCTs)	⨁⨁◯◯ Low[a,b,i,j]	-		MD **215.83 g higher** (87.33 higher to 344.34 higher)
Mean gestational age at birth - treatment trials	136 (3 RCTs)	⨁⨁◯◯ Low[a,b,d,f]	-		MD **1.01 weeks higher** (0.08 lower to 2.1 higher)

Secondary neonatal outcomes – treatment trials

L-arginine compared to placebo or no treatment for the treatment of preeclampsia in pregnant women

Patient or population: the treatment of preeclampsia in pregnant women

Intervention: L-arginine

Comparison: placebo or no treatment

Outcomes	№ of participants (studies) Follow-up	Certainty of the evidence (GRADE)	Relative effect (95% CI)	Anticipated absolute effects	
				Risk with placebo or no treatment	Risk difference with L-arginine
Neonatal mortality - treatment trials	157 (2 RCTs)	⊕◯◯◯ Very low[a,b,d,g]	**RR 0.36** (0.03 to 3.87)	26 per 1,000[#]	**17 fewer per 1,000** (25 fewer to 75 more)
Stillbirth - treatment trials	96 (1 RCT)	⊕◯◯◯ Very low[a,b,d,h]	**RR 0.92** (0.02 to 45.52)	0 per 1,000[#]	**0 fewer per 1,000** (0 fewer to 0 fewer)
Nitric oxide serum level - treatment trials	68 (1 RCT)	⊕⊕◯◯ Low[b,h]	-		MD **17.57 µmol/L higher** (7.73 higher to 27.41 higher)

***The risk in the intervention group** (and its 95% confidence interval) is based on the assumed risk in the comparison group and the **relative effect** of the intervention (and its 95% CI).
[#]The absolute risk difference should be interpreted with caution due to the small number of trials and few or no events in either group.
CI: confidence interval; **MD:** mean difference; **RR:** risk ratio

GRADE Working Group grades of evidence
High certainty: we are very confident that the true effect lies close to that of the estimate of the effect.
Moderate certainty: we are moderately confident in the effect estimate: the true effect is likely to be close to the estimate of the effect, but there is a possibility that it is substantially different.
Low certainty: our confidence in the effect estimate is limited: the true effect may be substantially different from the estimate of the effect.
Very low certainty: we have very little confidence in the effect estimate: the true effect is likely to be substantially different from the estimate of effect.

Explanations

a. Few events

b. There are not enough studies to test for publication bias

c. Strong association with RR less than 0.50

d. Wide confidence intervals and includes no effect

e. 1 out of 2 studies have a high risk of bias

f. 1 out of 3 studies have a high risk of bias

g. 2 out of 2 studies have high risk of bias

h. 1 of 1 study has high risk of bias

i. 2 out of 3 studies have a high risk of bias

j. Wide confidence interval
