# Supplementary material for: L‐Arginine and L‐Citrulline for Prevention and Treatment of Pre‐Eclampsia: A Systematic Review and Meta‐Analysis
Source: BJOG. 2025 Jan 12;132(6):698–708. doi: 10.1111/1471-0528.18070 (PMC11969923; doi:10.1111/1471-0528.18070)
Supplement: Supplementary file 5 — Data S1. [file BJO-132-698-s002.docx]

Table of Contents

[Table S1. List of registered trials that have not yet been completed and/or published 2](#_Toc184991082)

[Table S2. Examination of publication bias using Egger’s linear regression test and Begg’s rank correlation test 3](#_Toc184991083)

[Table S3. Characteristics of included non-randomized studies 4](#_Toc184991084)

[Characteristics of non-randomized interventional trials 6](#_Toc184991085)

[Findings from non-randomized interventional trials 6](#_Toc184991086)

[Table S4. Characteristics of randomized controlled trials 7](#_Toc184991087)

[Figure S1. Funnel plot for A) mean birth weight, B) mean gestational age at birth 12](#_Toc184991088)

[Figure S2. Risk of bias of included randomized controlled trials A) risk of bias domains B) overall risk of bias 13](#_Toc184991089)

[Figure S3 Forest plot for adverse events 14](#_Toc184991090)

[Figure S4. Forest plots of secondary fetal/neonatal outcomes A) small-for-gestational-age infants B) neonatal intensive care unit admissions C) mean birth weight D) mean gestational age at birth E) low birth weight <2500 g F) neonatal hypoglycemia G) respiratory distress syndrome H) neonatal infections 19](#_Toc184991091)

[Figure S5. Forest plots of subgroup analysis by risk categories of the included population A) mean birth weight B) mean systolic blood pressure C) mean diastolic blood pressure D) mean gestational age at birth 23](#_Toc184991092)

[Subgroup analysis by indication for trial entry 24](#_Toc184991093)

[Figure S6. Risk of bias of included non-randomized trials 25](#_Toc184991094)

[Figure S7. Forest plots of outcomes reported in non-randomized trials A) mean birth weight B) mean gestational age at birth C) respiratory distress syndrome D) neonatal infections E) caesarean section F) fetal growth restriction 27](#_Toc184991095)

[References 28](#_Toc184991096)

## Table S1. List of registered trials that have not yet been completed and/or published

| **Title of trial** | **Registration ID** | **Trial phase** | **Date of registration** | **Recruitment status** |
| --- | --- | --- | --- | --- |
| L-ArGinine to pRevent advErse prEgnancy Outcomes (AGREE) | NCT05934318 | Not applicable | 5 April 2023 | Not yet recruiting |
| L-citrulline supplementation to reduce adverse pregnancy outcomes | PACTR202303697293140 | Phase III | 22 March 2023 | Not yet recruiting |
| Comparison between L-arginine and aspirin and aspirin alone in preventing preeclampsia in high-risk pregnant women | IRCT20221108056440N1 | Phase III | 27 November 2022 | Recruiting |
| Effect of L-arginine versus control group on the prevention of preeclampsia in primiparous pregnant women | IRCT20120215009014N416 |  | 12 January 2022 | Recruiting |
| Role of L-citrulline in Prevention of Pregnancy Associated Hypertension | NCT04979793 | Phase I | 16 June 2021 | Suspended |
| Oral L-Citrulline and ADMA in Pregnancy | NCT00743210 | Phase I | 26 August 2008 | Completed |
| L-arginine Effects on Chronic Hypertension in Pregnancy | NCT00974714 | Phase III | 9 September 2009 | Completed |
| Effects of Oral L-Arginine on Chronic Hypertension in Pregnancy | NCT00571766 | Phase III | 11 December 2007 | Completed |

## Table S2. Examination of publication bias using Egger’s linear regression test and Begg’s rank correlation test

| Outcomes^a^ | Number of studies | P for Egger’s test | P for Begg’s test |
| --- | --- | --- | --- |
| Mean birth weight | 17 | 0.6355 | 0.3004 |
| Mean gestational age at birth | 15 | **0.0162** | 0.1982 |

^a^Only outcomes reported by at least 10 studies were examined for publication bias

## Table S3. Characteristics of included non-randomized studies

| **Study** | **Country** | **Country income level** | **Population** | **Study design** | **Gestational age** | **Sample size** | **Intervention group:**  **L-arginine dose and route of administration** | **Control group: route and/or form of administration** | **Duration of intervention** | **Outcomes of interest** |
| --- | --- | --- | --- | --- | --- | --- | --- | --- | --- | --- |
| Neri 2006 | Italy | High | Pregnant women with gestational hypertension without proteinuria | RCT | 24 – 36 weeks | 123 | 20g/500ml/day IV | Placebo: IV infusion | 5 days | BP (not included in the analysis because only change values were reported) |
| Neri 2004^1^ | Italy | High | Pregnant women with mild to moderate gestational hypertension | RCT | 30 – 34 weeks | 15 | Daily 20g/500ml in IV, over 40 min | IV saline infusion | 2 days | None |
| Dera 2007^2^ | Poland | High | Pregnant women with gestational hypertension or fetal growth restriction | Non-RCT | 25 – 34 weeks | 69 | 3g/day orally | Placebo: oral | Not stated | CS, RDS, IUGR, neonatal infection, birth weight, GA at birth |
| Germain 2001^3^ | Chile | High | Pregnant women with prior placentation-related disorders | Non-RCT | From 10 weeks | 15 | 0.1g/kg/day orally | Placebo: oral | 2 weeks | None |
| Kumar 2015^4^ | India | Lower-middle | Pregnant women with asymmetrical FGR | Non-RCT | 30 – 32 weeks | 100 | 5g/day orally | Historical control treated with bed rest at home | 28 days | CS, RDS, IUGR  neonatal infection, birth weight, GA at birth, NICU admissions, neonatal intracranial hemorrhage |
| Shweta 2014^5^ | India | Lower-middle | Pregnant women with IUGR | Non-RCT | 30 – 32 weeks | 20 | Not stated | Without nutritional supplementation | Not stated | Nitric oxide serum level |

FGR: fetal growth restriction; BP: Blood pressure; CS: cesarean section; NO: nitric oxide; GA: gestational age: SGA: small for gestational age; RDS: respiratory distress syndrome; NICU: neonatal intensive care unit

### Characteristics of non-randomized interventional trials

Of the five^1-5^ non-RCTs, three^2, 4, 5^ reported on review outcomes. They involved 189 women with sample sizes ranging from 20^5^ to 100.^4^ Two studies were conducted in India^4, 5^ and one in Poland.^2^ Two included women with IUGR^4, 5^ and one included women with HDP or IUGR^2^ (Supplementary Table S2). One study started supplementation in the second or third trimester (25 – 34 weeks)^2^ and two in the third trimester (30 – 32 weeks).^4, 5^ Women were supplemented for 4 weeks in one study,^4^ whereas the other two studies did not state the duration of supplementation.^2, 5^ L-arginine was administered orally at a dose of 3g/day^2^ and 5g/day^4^ in one study each whereas one study did not state the dose and mode of administration. The comparison group received placebo,^2^ no intervention^5^ or bed rest at home.^4^ All three non-RCTs were judged as having serious risk of bias (Supplementary Figure S4).

### Findings from non-randomized interventional trials

Findings from the non-RCTs are presented in Supplementary Figure S5. Mean differences for birth weight and gestational age at birth were MD 496.51 g (95% CI 259.50, 733.52) and MD 2.08 weeks (95% CI 1.18, 2.99) respectively, in women supplemented with L-arginine compared to placebo. Relative risks following L-arginine supplementation compared to placebo were cesarean section (RR 0.79; 95% CI 0.59, 1.05); IUGR (RR 1.00; 95% CI 0.73, 1.37); neonatal infection (RR 1.03; 95% CI 0.50, 2.10); and RDS (RR 0.38; 95% CI 0.24, 0.60).

## Table S4. Characteristics of randomized controlled trials

| **Study** | **Country** | **Income level** | **Population** | **Type of trial^a^** | **Gestational age at enrolment** | **Sample size** | **Intervention group:**  **dose and route of administration** | **Control group: route and/form of administration** | **Duration of intervention** | **Outcomes** |
| --- | --- | --- | --- | --- | --- | --- | --- | --- | --- | --- |
| Camarena Pulido 2016^6^ | Mexico | Upper-middle | Pregnant women with high-risk factors for developing preeclampsia^b^ | Prevention | 20 weeks | 100 | 3g/day orally | Placebo: oral | Not stated | Incidence of preeclampsia, severe preeclampsia, preterm birth, birth weight, CS, NICU admission, FGR, adverse effects |
| Facchinetti 2007^7^ | Italy | High | Pregnant women with gestational hypertension with or without proteinuria | Prevention and treatment | 24 – 36 weeks | 80 | 20g/500ml IV over 4 hours given daily for 5 days then 4g/day orally for 2 weeks | Placebo: saline infusion 500ml IV over 4 hours given daily for 5 days | 19 days | Incidence of preeclampsia, severe preeclampsia, preterm birth, CS, BP, birth weight, GA at delivery, adverse effects |
| Khansari 2024 | Iran | Upper-middle | First-time pregnant women with no chronic hypertension, thyroid conditions, diabetes, high BP, or infections | Prevention | 20 weeks | 160 | 1g/day + prenatal supplements | Prenatal supplements | 12 weeks | Incidence of preeclampsia, severe preeclampsia, preterm birth, CS, birth weight, GA at delivery, FGR, adverse effects |
| Neri 2008^8^ | Italy | High | Pregnant women with pre-existing hypertension in pregnancy | Prevention | 16 – 18 weeks | 62 | 4g/day orally | Observation only | Not stated | BP, birth weight,  GA at delivery |
| Neri 2010^9^ | Italy | High | Pregnant women with mild chronic hypertension or mildly high BP before the 20^th^ week | Prevention | <16 weeks | 80 | 4g/day orally | Placebo: oral tablets | 10 – 12 weeks | BP, preterm birth, NICU admissions, birth weight, GA at delivery |
| Ormesher 2024^10^ | UK | High | Pregnant women with chronic hypertension | Prevention | 12 – 16 weeks | 36 | 3g twice daily (30ml syrup) | Placebo: oral syrup | 8 weeks | Incidence of preeclampsia, BP, birth weight, SGA, FGR, GA at delivery |
| Ropacka 2007^11^ | Poland | High | Pregnant women with FGR | Prevention | 24 – 36 weeks | 41 | 3g/day orally | Placebo: oral tablets | Not stated | CS, FGR, RDS, neonatal infection, birth weight, NICU admissions, GA at delivery |
| Rytlewski 2008^12^ | Poland | High | Pregnant women presenting with preterm labour | Prevention | 25 – 34 weeks | 70 | 3g/day orally | Placebo: oral tablets | Until delivery or 35 weeks | FGR, birth weight, GA at delivery |
| Shen 2011^13^ | China | Upper- middle | Pregnant women with FGR | Prevention | Not stated | 60 | 15g/500ml 5% glucose/day IV + conventional treatment | Conventional treatment | Not stated | CS, SGA, birth weight |
| Sieroszewski 2004^14^ | Poland | High | Pregnant women with FGR | Prevention | Not stated | 108 | 3g/day orally | No intervention | 20 days | SGA (growth retarded newborn), birth weight, GA at delivery |
| Singh 2015^15^ | India | Lower-middle | Pregnant women with asymmetrical FGR | Prevention | 30 – 40 weeks | 60 | 3g/day orally | Placebo: oral tablets | 21 days | CS, NO serum level, NICU admissions, neonatal hypoglycemia, neonatal mortality, RDS, stillbirth, birth weight, GA at delivery |
| Vadillo-Ortega 2011/2012^16, 17^ | Mexico | Upper- middle | Pregnant women with increased risk of preeclampsia^c^ | Prevention | 14 – 32 weeks | 672 | 6.6g/day (in two bars) + antioxidant vitamins orally | Antioxidant vitamins | Until delivery | Preterm birth, incidence of preeclampsia or eclampsia, GA at delivery, birth weight, CS |
| Vinitha 2018^18^ | India | Lower-middle | Pregnant women with FGR | Prevention | Not stated | 60 | 3g/day orally + routine therapy | Routine therapy | 4 weeks | Stillbirth, birth weight, CS, NICU admissions, GA at delivery, RDS, neonatal hypoglycemia |
| Winer 2009^19^ | France | High | Pregnant women with FGR | Prevention | 24 – 32 weeks | 47 | 14g (90cc)/ day in two equal doses of oral syrup | Placebo: oral syrup | Until delivery | BP, birth weight, NO serum levels, GA at delivery |
| Xiao 2005^20^ | China | Upper- middle | Pregnant women with asymmetric FGR | Prevention | Not stated | 66 | 20g/day IV + routine therapy | Routine therapy | 7 days | NO plasma levels, GA at delivery, birth weight |
| Zhang 2007^21^ | China | Upper- middle | Pregnant women with hypertensive disorders in pregnancy and FGR | Treatment | Not stated | 68 | 20g/day IV + conventional treatment | Conventional treatment | Not stated | Birth weight, NO serum levels |
| Hladunewich 2006^22^ | USA | High | Pregnant women with preeclampsia | Treatment | Not stated | 45 | 3.5g every 6 hours orally or 10g every 8 hours IV (when could not be taken orally) | Placebo: oral or IV (when could not be taken orally) | Up to 3 days postpartum | BP, GA at delivery |
| Rytlewski 2005^23^ and secondary analysis Rytlewski 2006^24^ | Poland | High | Pregnant women with preeclampsia and no history of chronic hypertension | Treatment | 26 – 34 weeks | 83 | 3g/day orally | Placebo: oral tablets | 3 weeks/ until delivery | BP, Neonatal mortality, FGR  birth weight, GA at delivery |
| Staff 2004^25^ | Norway | High | Pregnant women with preeclampsia | Treatment | 28 – 36 weeks | 30 | 4g three times/day orally | Placebo: oral tablets | Up to 5 days (less for those who delivered earlier) | BP, birth weight, GA at delivery, NICU admissions, CS |
| Valdivia-Silva 2009^26^ | Peru and Mexico | Upper- middle | Pregnant women with preeclampsia | Treatment | Not stated | 100 | 1g three times/day orally | Placebo: oral tablets | >3 weeks and until delivery | FGR, neonatal mortality, stillbirth |

^a^Prevention trials were those that included women before the onset of pre-eclampsia (regardless of risk) and treatment trials included women diagnosed with preeclampsia according to ISSHP definition; ^b^Such as nulliparous, previous history of preeclampsia, chronic hypertension, and body mass index [BMI] ≥ 30; ^c^Defined as either a personal history of pre-eclampsia or pre-eclampsia in a first degree relative; FGR: fetal growth restriction; BP: blood pressure; CS: cesarean section; NO: nitric oxide; GA: gestational age; SGA: small for gestational age; RDS: respiratory distress syndrome; NICU: neonatal intensive care unit

**A**

**B**

## Figure S1. Funnel plot for A) mean birth weight, B) mean gestational age at birth

**
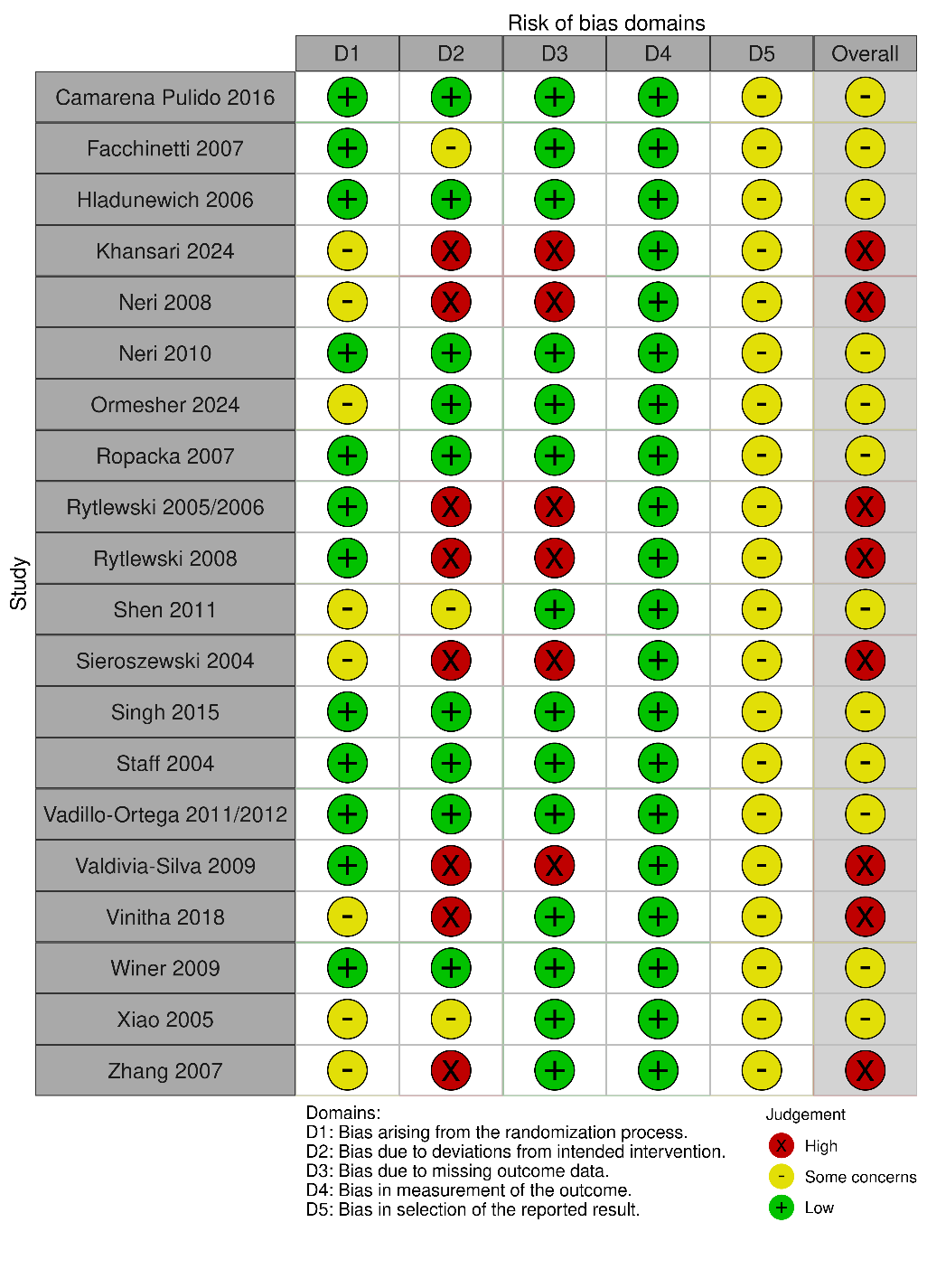
**

**B**


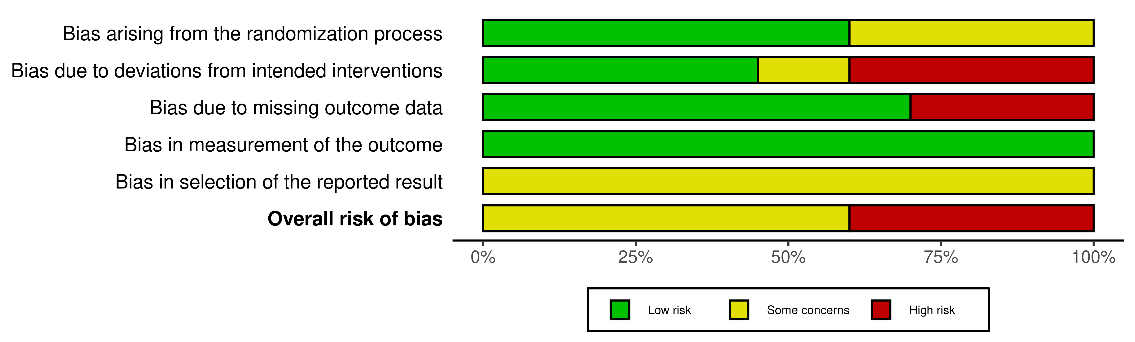


## Figure S2. Risk of bias of included randomized controlled trials A) risk of bias domains B) overall risk of bias

## Figure S3 Forest plot for adverse events

**A**

**B**

**C**

**D**

**E**

**F**


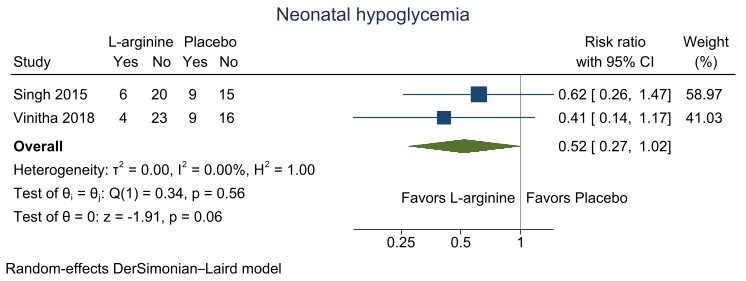


**G**

**H**

## Figure S4. Forest plots of secondary fetal/neonatal outcomes A) small-for-gestational-age infants B) neonatal intensive care unit admissions C) mean birth weight D) mean gestational age at birth E) low birth weight <2500 g F) neonatal hypoglycemia G) respiratory distress syndrome H) neonatal infections

**A**

**B**

**C**

**D**

## Figure S5. Forest plots of subgroup analysis by risk categories of the included population A) mean birth weight B) mean systolic blood pressure C) mean diastolic blood pressure D) mean gestational age at birth

### Subgroup analysis by indication for trial entry

In subgroup analysis based on the indication for trial entry (Supplementary Figure S3), L-arginine may increase mean birth weight in women with HDP and FGR (MD 200.00 g; 95% CI 57.33, 342.67; one trial; 35 women)^21^ and in women with FGR (MD 182.18 g; 95% CI 56.33, 308.04; 16 trials; 243 women).^11, 13-15, 18-20^ In women with other HDPs or at high risk of preeclampsia, L-arginine may decrease systolic (MD -3.69 mmHg; 95% CI, -6.47, -0.09; three trials; 104 women) and diastolic BP (MD -3.74 mmHg; 95% CI, -6.36, -1.12; three trials; 104 women)^7-9^ and increase mean gestational age at birth (MD 0.70 weeks; 95% CI 0.16, 1.25; four trials; 332 women).^7-9, 17^ In women diagnosed with preeclampsia, systolic BP may decrease following L-arginine supplementation (MD -5.64 mmHg; 95% CI, -10.66, -0.62; three trials; 136 women).^22, 23, 25^


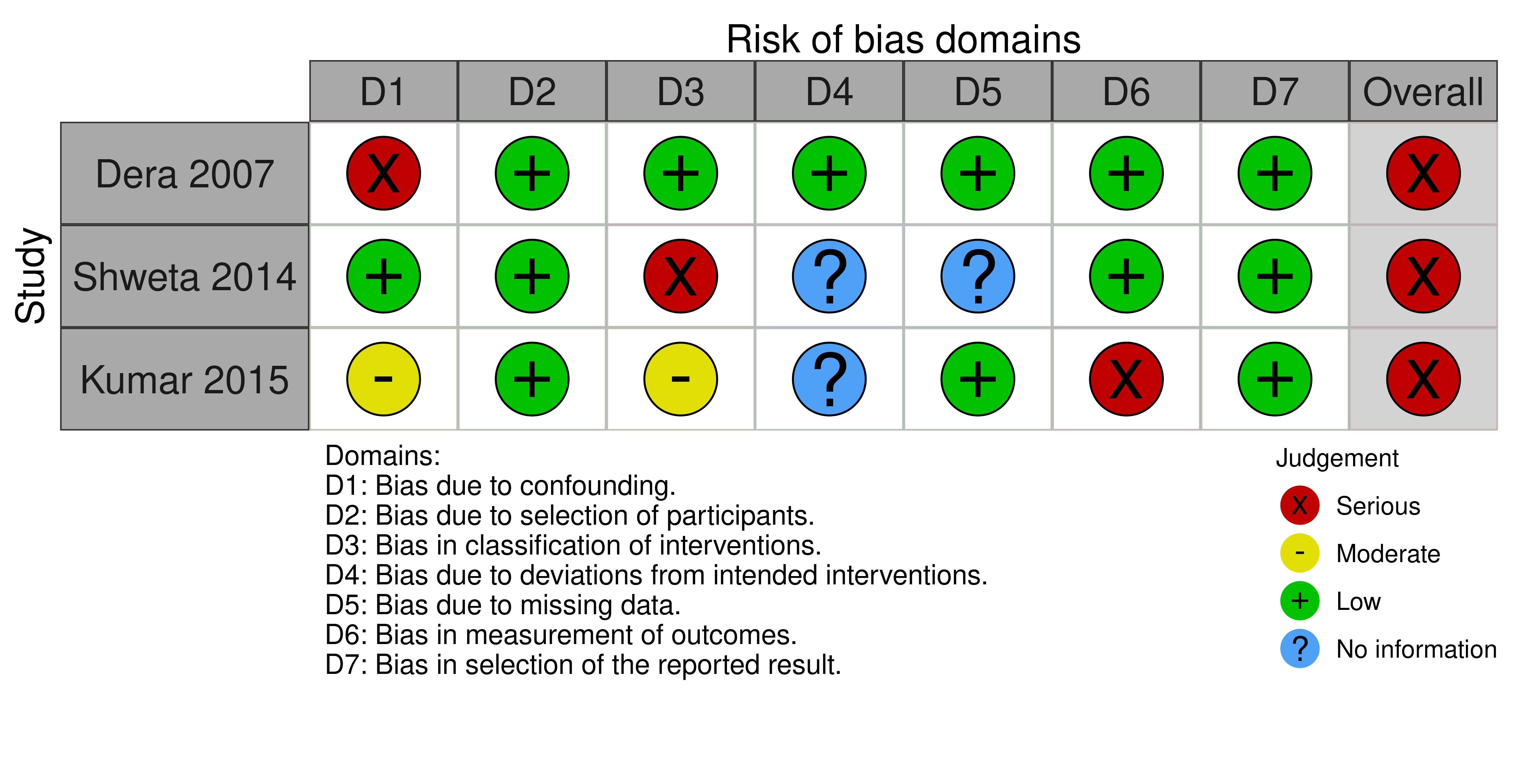

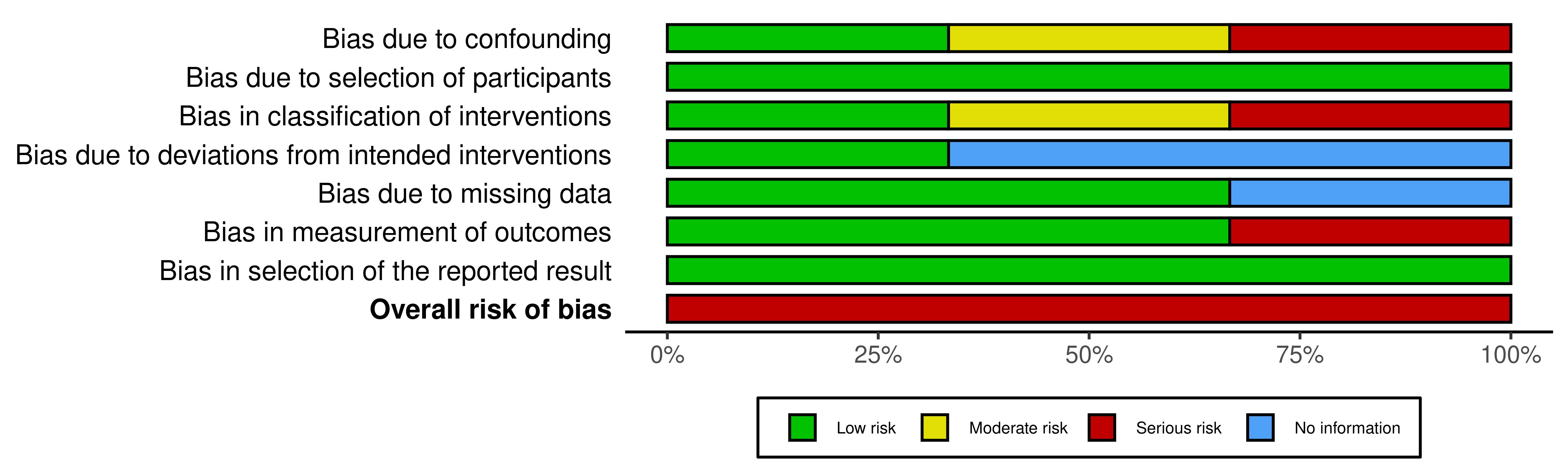


## Figure S6. Risk of bias of included non-randomized trials

**A**

**B**

**C**

**D**

**E**

**F**

## Figure S7. Forest plots of outcomes reported in non-randomized trials A) mean birth weight B) mean gestational age at birth C) respiratory distress syndrome D) neonatal infections E) caesarean section F) fetal growth restriction

## References

1. Neri I, Blasi I, Facchinetti F. Effects of acute L-arginine infusion on non-stress test in hypertensive pregnant women. Journal of maternal-fetal & neonatal medicine. 2004;Vol.16(1):23-6p.

2. Dera A, Ropacka M, Kowalska J, Markwitz W, Nycz P, Breborowicz G. The effect of L-arginine treatment on the neonatal outcome from pregnancies complicated by intrauterine growth restriction and gestational hypertension. Arch perinat med. 2007;13(3):35-9.

3. Germain Aravena A, Irribarra P V, Faúndez Giordano L, Valdés Stromilli G. ¿Puede prevenirse la preeclampsia a través del reconocimiento de la disfunción endotelial primaria y de su manejo con L-Arginina?: ¿cómo ayuda el ultrasonido Doppler? Rev chil ultrason. 2001;4(3):72-8.

4. Kumar MA, Jayati M, Rudrajit P. Comparative study of oral L-arginine therapy versus non-ambulatory treatment in pregnancy with asymmetrical fetal growth restriction. International research journal of pharmacy. 2015;Vol.6(6):371-3p.

5. Shweta, Anuradha K. The Effect of Antenatal L-Arginine and Antioxidant Supplementation n Oxidative Stress Marker Levels in Newborns. Journal of Clinical and Diagnostic Research. 2014;8(10):OC10-OC2.

6. Camarena Pulido EE, Garcia Benavides L, Panduro Baron JG, Pascoe Gonzalez S, Madrigal Saray AJ, Garcia Padilla FE, et al. Efficacy of L-arginine for preventing preeclampsia in high-risk pregnancies: A double-blind, randomized, clinical trial. Hypertension in Pregnancy. 2016;35(2):217-25.

7. Facchinetti F, Saade GR, Neri I, Pizzi C, Longo M, Volpe A. L-arginine supplementation in patients with gestational hypertension: a pilot study. Hypertension in pregnancy. 2007;Vol.26(1):121-30p.

8. Neri I, Monari F, De Pace V, Facchinetti F, Volpe A. L-arginine and hypertension in pregnancy. [Italian]. Giornale Italiano di Ostetricia e Ginecologia. 2008;30(4):139-41.

9. Neri I, Monari F, Sgarbi L, Berardi A, Masellis G, Facchinetti F. L-arginine supplementation in women with chronic hypertension: impact on blood pressure and maternal and neonatal complications. Journal of maternal-fetal & neonatal medicine. 2010;Vol.23(12):1456-60p.

10. Ormesher L, Worton SA, Best A, Dodd SR, Dempsey A, Cottrell EC, et al. CHronic hypERtension and L-citRulline studY (CHERRY): an Early-Phase Randomised Controlled Trial in Pregnancy. Reprod Sci. 2024;31(2):560-8.

11. Ropacka M, Kowalska J, Hepner K, Markwitz W, Breborowicz G. The effect of L-arginine on fetal outcome in IUGR fetuses. Arch Perinat Med. 2007;13(3):30-4.

12. Rytlewski K, Olszanecki R, Lauterbach R, Grzyb A, Kiec-Wilk B, Dembinska-Kiec A, et al. Effects of oral L-arginine on the pulsatility indices of umbilical artery and middle cerebral artery in preterm labor. European journal of obstetrics, gynecology, and reproductive biology. 2008;Vol.138(1):23-8p.

13. Shen SF, Hua CH. Effect of L-arginine on the expression of Bcl-2 and Bax in the placenta of fetal growth restriction. Journal of maternal-fetal & neonatal medicine. 2011;Vol.24(6):822-6p.

14. Sieroszewski P, Suzin J, Karowicz B, nacute;ska A. Ultrasound evaluation of intrauterine growth restriction therapy by a nitric oxide donor (L-arginine). Journal of maternal-fetal & neonatal medicine. 2004;Vol.15(6):363-6p.

15. Singh S, Sharma D, Singh A, Narula MK, Bhattacharjee J. Effect of l-Arginine on Nitric Oxide Levels in Intrauterine Growth Restriction and its Correlation with Fetal Outcome. Indian Journal of Clinical Biochemistry. 2015;30(3):298-304.

16. Vadillo F, Perichart O, Espino S, Avila MA, Ibar. Randomised clinical study of the effect of dietary supplementation during pregnancy with L-arginine and antioxidant vitamins in preeclampsia in a high-risk population. Revista chilena de obstetricia y ginecolog&iacute;a. 2012;Vol.77(3):243-5p.

17. Vadillo-Ortega F, Perichart-Perera O, Espino S, Avila-Vergara MA, Ibarra I, Ahued R, et al. Effect of supplementation during pregnancy with L-arginine and antioxidant vitamins in medical food on pre-eclampsia in high risk population: randomised controlled trial. BMJ. 2011;342:d2901.

18. Vinitha PM, Bareen HA, Padmanaban S. L-Arginine supplementation in iugr and its effect on fetal outcome: A randomised control trial. International Journal of Clinical Obstetrics and Gynaecology. 2018;2(6):114-7.

19. Winer N, Branger B, Azria E, Tsatsaris V, Philippe HJ, Roze JC, et al. L-Arginine treatment for severe vascular fetal intrauterine growth restriction: a randomized double-blind controlled trial. Clinical nutrition (Edinburgh, Scotland). 2009;Vol.28(3):243-8p.

20. Xiao XM, Li LP. L-arginine treatment for asymmetric fetal growth restriction. International journal of gynaecology and obstetrics. 2005;Vol.88(1):15-8p.

21. Zhang N, Xiong AH, Xiao X, Li LP. Effect and mechanism of L-arginine therapy for fetal growth retardation due to pregnancy-induced hypertension. [Chinese]. Nan fang yi ke da xue xue bao = Journal of Southern Medical University. 2007;27(2):198-200.

22. Hladunewich MA, Derby GC, Lafayette RA, Blouch KL, Druzin ML, Myers BD. Effect of L-arginine therapy on the glomerular injury of preeclampsia: a randomized controlled trial. Obstetrics and gynecology. 2006;Vol.107(4):886-95p.

23. Rytlewski K, Olszanecki R, Korbut R, Zdebski Z. Effects of prolonged oral supplementation with l-arginine on blood pressure and nitric oxide synthesis in preeclampsia. European Journal of Clinical Investigation. 2005;35(1):32-7.

24. Rytlewski K, Olszanecki R, Lauterbach R, Grzyb A, Basta A. Effects of oral L-arginine on the foetal condition and neonatal outcome in preeclampsia: a preliminary report. Basic & clinical pharmacology & toxicology. 2006;Vol.99(2):146-52p.

25. Staff AC, Berge L, Haugen G, Lorentzen B, Mikkelsen B, Henriksen T. Dietary supplementation with L-arginine or placebo in women with pre-eclampsia. Acta obstetricia et gynecologica Scandinavica. 2004;Vol.83(1):103-7p.

26. Valdivia-Silva JE, Lopez-Molina K, Macedo R. Effect of early L-arginine therapy on intrauterine growth restriction in preeclampsia. A randomized controlled trial in Latin-American women. Progresos en obstetricia y ginecologia. 2009;Vol.52(2):89-98p.
